# Assisted total thermal ablation: presentation of the ATTA technique

**DOI:** 10.1590/1677-5449.202200481

**Published:** 2022-11-14

**Authors:** Daniel Amatuzi, Daniel Autran Burlier Drummond, Douglas Poschinger-Figueiredo, Lucas Barbosa-Silva, Julio Cesar Peclat de Oliveira, Marcos Arêas Marques

**Affiliations:** 1 Clínica Vascular ATTA Concept, Maringá, PR, Brasil.; 2 Clínica Daniel Drummond, Rio de Janeiro, RJ, Brasil.; 3 Universidade do Estado do Rio de Janeiro - UERJ, CT Vascular, Rio de Janeiro, RJ, Brasil.; 4 Clínica Patrícia Holderbaum, Canoas, RS, Brasil.; 5 Clínica Peclat, Rio de Janeiro, RJ, Brasil.; 6 Universidade do Estado do Rio de Janeiro - UERJ, Rio de Janeiro, RJ, Brasil.; 7 Universidade Federal do Estado do Rio de Janeiro - UNIRIO, Rio de Janeiro, RJ, Brasil.

**Keywords:** varicose veins, venous insufficiency, saphenous vein, laser treatment, quality of life, esthetics

## Abstract

Treatment of lower limb chronic venous disease has progressed exponentially over recent decades. The advances achieved have made it possible to develop a proposal for a systematized intravenous laser ablation technique — assisted total thermal ablation (ATTA). The technique constitutes a standardized method for management of axial or tributary veins that are varicosed or esthetically unappealing, whether in the lower limbs or other areas, that can be performed on an outpatient or day-hospital basis. This article describes the processes for preoperative preparation and detailed marking, the materials needed, venous access, anesthesia, calculation of power and energy, the ablation technique itself, follow-up, and adverse events. The ATTA technique is proposed as a tool for treatment of chronic venous disease and of esthetically unappealing veins, suggesting possible extension of the applications for lasers beyond trunk veins to any vein that can be punctured.

## INTRODUCTION

Treatment of lower limb chronic venous disease (CVD) has progressed exponentially over recent decades, transitioning from the classic techniques to newer less invasive methods

Ablative endovascular techniques have gained particular prominence, with a tendency to move out of hospitals and increasingly employ exclusively ambulatory settings for procedures. One of the most versatile and widely-used techniques is endovenous laser therapy (EVLT).^1^^,^^2^

After the first case of axial treatment was described in 1998 by Boné,^3^ many different improvements and optimizations of the method have been developed; such as transition from flat to radial fibers with smaller calibers, development of laser generators with better energy delivery profiles, and extension of their applications to use in tributary veins.^4^^,^^5^

The advantages of thermoablative techniques include shorter duration of convalescence and of the need to wear elastic compression after the procedure, resulting in increased patient convenience. There is also less injury to perivenous tissues, lower risk of bleeding and infection, and the possibility of performing the procedure without an anesthetist, with equivalent efficacy, safety, and occlusion rates to conventional surgery.^6^^-^9

Although EVLT is the technique of choice for treatment of axial veins of the superficial vein system, according to both the Society for Vascular Surgery and the European Society for Vascular Surgery, the same societies recommend ambulatory phlebectomy for treatment of varicose tributaries.^10^^,^^11^

The predominant conduct in Brazil is to perform multiple phlebectomies in a hospital setting, normally under spinal anesthesia or general anesthesia. An alternative option is ambulatory phlebectomy, which is grossly underutilized, due to limitations in cases with widespread disease, which is frequently observed in Latin America.^12^

In this innovation article, we describe a proposal for a standardized EVLT technique, assisted total thermal ablation (ATTA), and report on perioperative adverse events observed and the technique’s safety profile.

## METHODS

The operating technique is described in detail, clearly explaining the steps involved and the logistic aspects of execution, in order to enable it to be replicated.

The technique consists of treatment of trunk and/or tributary veins with endolaser in an ambulatory or day hospital setting. Some patients will require supplementary treatment during follow-up. On a case-by-case basis, depending on esthetic needs, transdermal laser or liquid sclerotherapy can be used to supplement the initial treatment.

Patients are considered candidates for treatment with this technique if they have veins that are incompetent, dilated, tortuous, varicosed, or esthetically unappealing in the lower limbs or other areas, including upper limbs and face. In addition to the saphenous axes, the technique is also applicable to tributaries that connect trunk veins to reticular veins and telangiectasias.^10^^,^^11^

The following were defined as contraindications to treatment: active cutaneous infection in the area to be treated; American Society of Anesthesiologists (ASA) surgical risk grade III or IV; patient on anticoagulants or with hypercoagulable state; pregnancy or postpartum; and history of allergy to the drugs used when performing the technique.

### Technical protocol

After taking the candidate patient’s history and performing a physical examination, a phlebogram is performed. This consists of using vascular ultrasonography with Doppler (USD) to map the venous system in detail, recording parameters of interest for conducting the ATTA technique, including the paths, locations, and calibers of the vessels to be treated and the distance from the vein wall to the skin and proximity to adjacent structures.

1. Detailed marking
  - Room temperature maintained at 25 ºC, to avoid vasoconstriction in response to cold;
  - Photographic record prepared of the areas to be treated in all views, including images with the patient standing, prone, and supine;
  - Provision of the phlebogram conducted previously;
  - Arrangement of equipment for ultrasonography, vein transillumination, and/or augmented reality, to check calibers and paths of trunk veins, tributaries, and perforating veins; and
  - Marking of veins of interest, followed by photographic record. A set of symbols for marking is suggested in Table 1.

**Table 1:** Symbols used for detailed marking of the planned treatment paths.

**Symbols**	**Interpretation**
- - - - - - - - - - -	Path of dysfunctional saphenous vein.
broken line	
. . . . .	Planned sites of anesthetic blebs and insertion of tumescent anesthesia needles or intravenous catheters.
dots	
→ → → → → →	Planned path of advance of catheters, after puncture of the vein.
directional arrows	
+ + +	Planned sites for insertion of catheters.
crosses

In the first step, using the phlebogram for reference, the patient is placed in the supine and prone positions and the paths of the trunk and/or tributary veins to be treated are marked out, with the aid of USD, the vein transilluminator, and/or augmented reality equipment.

**Figure 1:**
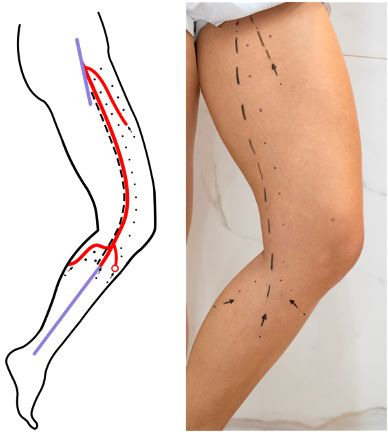
Detailed marking on the medial aspect of the left lower limb. **(A)** Phlebogram illustrating the ostial reflux in the internal saphenous and anterior accessory saphenous veins, transmitted to the anterior and posterior arcuate veins; **(B)** Standardized symbols suggested for planning the procedure.

During this step, possible sites for tumescence can be marked with dots and sites for insertion of catheters can be marked with dots and arrows (Figure 1).

The second step is performed with the patient standing upright, preferably on a raised platform. The markings are checked with ultrasound, confirming the paths of the saphenous veins and the sites for tumescence.

The tumescence sites are spaced 5 to 6 cm apart and follow the paths traced, longitudinally, at a distance of 2 to 3 cm from the path. Anesthetic blebs should be administered at these points and at the sites chosen for catheter insertion, ensuring patient comfort. Marking can be performed at the consultation the day before the procedure, or a short time before it.

1. Asepsis, materials, and anesthesia

The patient is placed in the supine position for conscious sedation, followed by analgesia with nitrous oxide. Antisepsis is performed with chlorhexidine 2% and sterile surgical fields are positioned, after setting up the instrument cart with the materials.^13^

**Figure 2:**
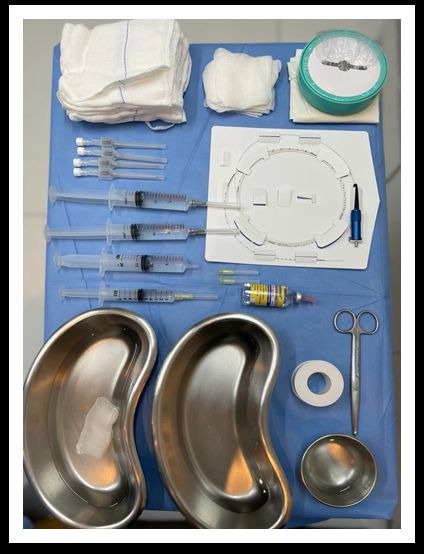
Materials needed for the assisted total thermal ablation (ATTA) technique.

The following instruments and materials are needed: a 1,470 nm endolaser machine; 400 μm and/or 600 μm radial fibers, the last of which is optional, to be used for saphenous veins with larger calibers; 6F x 11 cm introducer kit and/or intravenous 16G or 14G catheters for puncturing tributaries and saphenous veins; luer slip syringes for salination of accesses and 20 mL luer lock syringes for the tumescent solution; and a 10 mL luer lock syringe for administering anesthetic blebs (Figure 2).

The anesthetic blebs are administered with 0.2 to 0.3 mL per bleb of 1 to 2% lidocaine solution, without a vasoconstrictor, observing the 3 to 5 minutes anesthetic latency time.

1. Accesses

**Figure 3:**
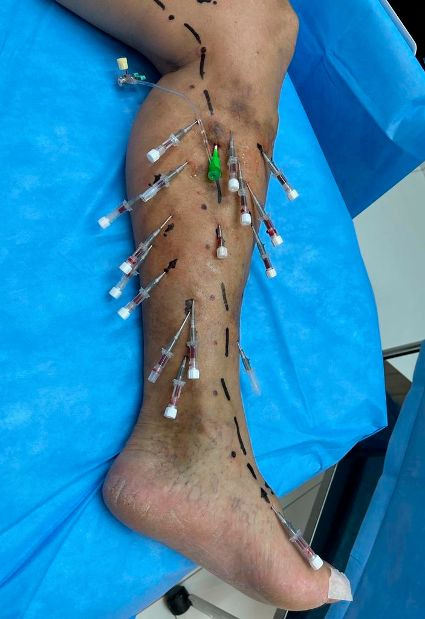
Left lower limb. Observe the use of standardized symbols for the technique. A 6F introducer kit is inserted into the internal saphenous vein and several catheters provide access to the tributary veins.

When there are indications to treat both axial and tributary veins in the same procedure, a logical sequence is essential to avoid vasoconstriction of the veins that are being accessed. Access to the internal saphenous is obtained first, preferably with the 6F introducer and then accesses to the tributaries are obtained (Figure 3).

The medial aspects of the foot, leg, and thigh are approached sequentially, followed by the lateral aspects of the foot, leg, and thigh. These veins are ablated in the same order proposed for access and, finally, the great saphenous is treated. Depending on the indications of each case, the patient is placed in the prone position to treat the small saphenous veins and posterior tributaries, following the protocol described.

The ideal point for puncture is 10 mm distal of the target vein, aiming to both enable complete ablation of the segment accessed, ensuring distance from the skin, and provide an anchorage point for the catheter, ensuring the safety of tumescence and thermoablation. The angle between needle and skin during puncture of tributaries are habitually less than 45º and needles are advanced parallel to the skin.

Utilizing the markings applied previously, it is suggested that as punctures are made, cross-sectional ultrasound images should be obtained of the vein and the intravenous catheter. The catheter tip should be maintained in the center of the vein, forming a “target” image. The catheter is advanced along the path of the vein, following its anatomy and navigating its tortuosity, to the length planned for treatment. Additional punctures will be needed when the angle of the vein provokes contact between the catheter and the vein wall, preventing it from advancing and risking damage to the vein.

The patient is placed in the Trendelenburg position to empty the venous bed. The catheter is salinated immediately after removal of the needle, with infusion of 2 to 3 mL of saline solution.

To treat the tributaries, habitually, the 400 μm radial fiber is delicately inserted via the catheters up to its maximum point of advancement. Even small vein segments should be treated in order to achieve the result. According to the “heat pipe” hypothesis, direct heat transfer and the optical-thermal effect take place approximately 6 mm distal of the fiber and temperatures of 100 ºC can be reached for a further 14 mm, via superheated bubbles, constituting an effective zone of thermal venous damage up to 20 mm beyond the final point at which the ablative instrument is positioned.^14^

1. Tumescence

The objectives of tumescence are hydrodissection, protection of perivenous structures, primarily the skin, and compression and constriction of the vein to be treated, reducing the quantity of blood in the lumen as much as possible, which lowers the rate of complications and amplifies the results achieved.^15^

The tumescent anesthetic solution of lidocaine 0.05%, saline, sodium bicarbonate, and adrenaline is used for all cases, even if the patient has been given a spinal block or general anesthesia, since it improves post-treatment comfort.^16^^,^^17^

The volume and concentration of lidocaine can be adjusted for the weight of the patient and the extent of the area to be treated, respecting the substance’s toxic dosage limits. The extremities are more sensitive to pain, as are areas adjacent to perforating veins, because of the proximity of neurovascular bundles.

**Figure 4:**
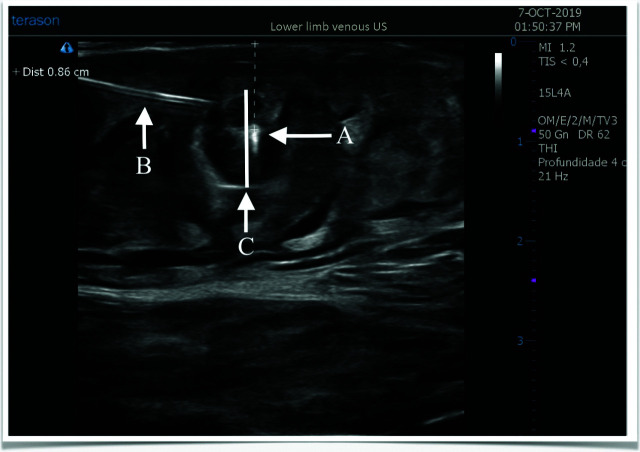
Ultrasonographic images acquired during tumescent anesthesia. Observe in the image: **(A)** the endolaser fiber; **(B)** the tumescent anesthesia needle; and **(C)** the tumescence halo, confirming the distance from the anterior vein wall to the skin.

One of the objectives of tumescence is to obtain a halo with a 10 mm radius circumjacent to the target vein, using the distance from the skin to the anterior vein wall as a reference, which is not always possible to achieve. If the tumescent halo is smaller than 8 mm, the calculation of how much power to utilize is affected (Figure 4).^15^^,^^16^

When treating areas where there are several punctures close to each other, it is recommended that, to reduce the risk of complications, the tumescent solution should be administered at a low temperature, of approximately 4 °C, and longer intervals of time should be allowed to elapse between successive ablations, to avoid creating a rising temperature gradient.

1. Calculating ablation energy and power

This is an extremely important step in the procedure. Thermal ablation raises concerns with regard to how much energy should be used because of the risk of complications, such as thermal injuries to the skin and other perivenous structures. The variables of relevance are the wavelength of the laser employed; the mode of energy delivery; the velocity of fiber traction (pullback); the type of fiber; the linear intravenous energy density (LEED); and the nominal power rating.^6^^,^^18^

A 1,470 nm diode laser offers the greatest advantages for intravenous thermoablation and there are publications demonstrating that it has a better energy delivery profile and superior treatment results in relation to other wavelengths.^4^^,^^19^

Endovenous laser equipment can delivery light energy in pulsed or continuous mode. The continuous mode offers easier quantification of the total energy delivered per vein segment treated, or by surface, and is the mode chosen for the ATTA method. Power delivery ranges from 1 to 40 watts (W), depending on the manufacturer of the unit.^20^

There is a wide-ranging debate on the best method for calculating the ideal energy to achieve effective thermal ablation when treating the main trunk veins. However, the most widely adopted method in Brazil was proposed in 2005 and consists of calculating the energy administered in the form of LEED, using the simplified formula LEED = P x T / linear centimeters of vein treated, where P is power and T is time.^21^

We therefore have two variables to deal with in the LEED calculation: the nominal power, in W, and the pullback velocity, in millimeters per second. In order to standardize the technique, we maintain pullback velocity at 1 mm/s.^21^

For the ATTA technique, we have developed a detailed protocol for calculating the power. Since the pullback velocity is fixed at 1 mm/s, the final LEED, in J/cm, is equivalent to ten times the nominal power setting.

**Figure 5:**
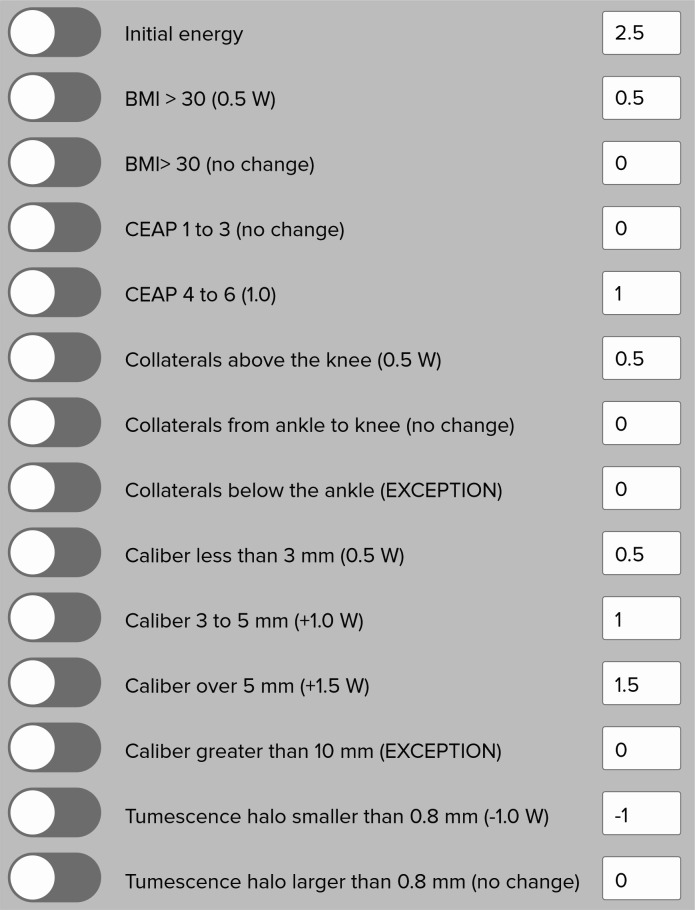
Translation of the interface of the ATTA Score supporting app (originally in Portuguese), for calculating the power rating to be used in the intervention. BMI = body mass index; CEAP = Clinical, Etiological, Anatomic, and Pathological classification.

The energy calculation should be performed when the procedure is being planned, normally after conducting the phlebogram, facilitating execution and speeding up the intervention (Table 2). Applications for mobile devices such as the ATTA Score can simplify the calculation (Figure 5). Measurement of the radius of the tumescent halo is a variable that has an impact on the calculation and must be determined during the procedure.

**Table 2:** Variables involved in power calculation.

**Variables**	Additional energy*
Initial energy level	2.5
Body mass index	
• > 30 kg/m^2^	0.5
• < 30 kg/m^2^	0
CEAP classification†	
• 1-3	0
• 4-6	1.0
Location of varicose veins	
• Tributaries above the knee	0.5
• Tributaries in the leg	0
• Tributaries in the ankle and/or below (exception)	0
Diameter of target vein	
• Up to 3 mm	0.5
• 3.1-5 mm	1.0
• 5.1-10 mm	1.5
• > 10 mm (exception)	0
Tumescence halo	
• < 8 mm	-1.0
• > 8 mm	0

* Energy in watts (W);

† Clinical, etiological, anatomic, and pathological (CEAP) clinical stage.

We start with a power rating of 2.5 W, which we then increase or reduce according to the following variables.

- Body mass index (BMI). When BMI is greater than or equal to 30 kg/m^2^, we increase power by 0.5 W, given the greater likelihood of recanalization after EVLT;^22^
- Clinical, etiological, anatomic, and pathological (CEAP) classification. We increase the power emitted by 1.0 W for patients at stages C4, C5, or C6 - since it has been demonstrated that reinterventions are more likely to be needed;^23^
- Location of the target vessel. Varicose veins in the thigh and knee are treated with an additional 0.5 W;
- Vein diameter. This variable is highly relevant to execution of the technique. For veins with calibers less than 3 mm, 0.5 W are added. From 3 to 5 mm in diameter, 1.0 W is added, and above 5 mm, 1.5 W are added to the power rating;^24^
- Tumescent anesthesia. This plays an important role in preventing complications, by creating a tumescent halo.

For tributaries below the knee, there is no addition or subtraction. Varicose veins in the ankles and feet constitute an exception to the calculation because they require different power ratings and pullback velocities. It is recommended that these veins should be treated by someone with greater technical experience, because more distal sites are associated with a greater risk of complications.

Perforating veins need higher energy quantities for adequate thermal ablation. Habitually, we add 1.0 W to the resulting power after using the calculation table.^25^

1. Ablation technique

After the power setting has been calculated and programmed on the laser equipment, we proceed to partially retract the catheter sheath and sequentially fire the laser. Pullback velocity should be maintained at a constant 1 mm/s, synchronizing the movement to withdraw the fiber with the audible signal of one beep per second, which is a function that is available on some laser machines. During thermal ablation, one hand tractions the fiber, while the other controls the transducer, observing ultrasonographic signs of the tissue response and monitoring any possible need for additional tumescence. During the final two centimeters, it becomes more practical to employ manual compression, maintaining the vein collapsed and monitoring the local temperature. The final steps consist of removal of the fiber together with the catheter sheath and application of digital compression for a minimum of 30 seconds, or until hemostasis is achieved.^14^

During the immediate control ultrasound examination, occlusion of the treated vein is noted and absence of flow is seen on USD. In a manner analogous to treatment of saphenous trunk veins, we can sometimes detect the “pearl sign” in cross-sectional images, a white line in the longitudinal plane, and also a mild acoustic shadow if bubbles have formed.^16^

**Figure 6:**
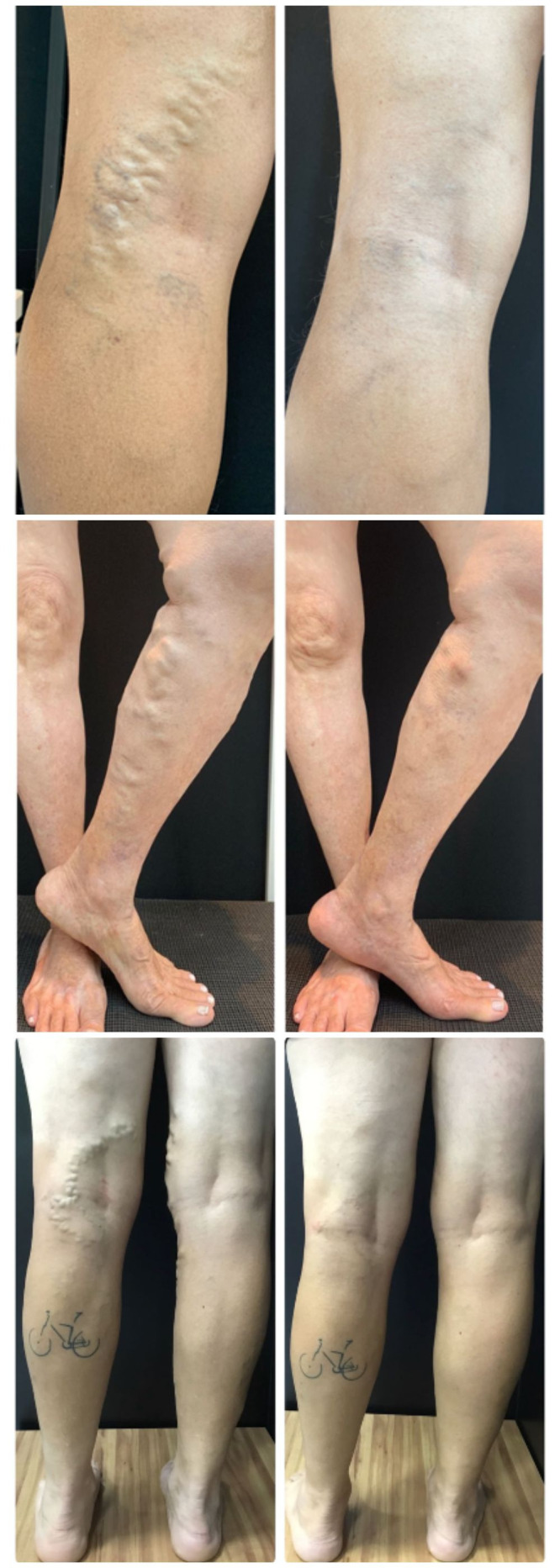
Pre and postoperative photographs of patients treated with the assisted total thermal ablation (ATTA) technique.

Dressings are applied with eccentric compression at the puncture sites, followed by elastic compression, habitually applied by 35 mmHg stockings for 24 h, with the objective of reducing edema and hematoma. The patient is instructed to wear the same 35 mmHg stockings or stockings providing a lower level of compression, from 20 to 30 mmHg, for 1 to 3 weeks during the daytime. Postoperative photographs are taken to provide a record that can be used as a reference for follow-up (Figure 6).^26^

Using the methodology described above, the ATTA technique can be applied to other parts of the body, such as to treat esthetically unappealing veins in the hands and forehead. These areas demand more precise execution, individualized care, and the local anatomy must be respected during tumescence and thermoablation.

1. Ultrasonographic follow-up

It is recommended that a control USD examination be performed between the third and seventh days after the procedure to rule out deep venous thrombosis (DVT) or endothermal heat induced thrombosis (EHIT), primarily in patients whose saphenous trunks have been treated where there is communication with perforating veins.^27^

### Safety and adverse events profile

Adverse events observed included pain, thrombophlebitis, ecchymosis, hyperchromia, induration, peripheral neurological injury, with transitory dysesthesia, and first degree skin burns. These events are also observed in association with other thermoablative techniques, and symptomatic cases were managed with non-steroidal anti-inflammatories for 5 days. With the exception of the hyperchromia and residual induration, the symptoms related to these adverse events were absent at 30 days.^7^^,^^16^

While they have been described in the literature, in our practice we have not observed sickness; rash; allergy; cervical constriction; coughing; thoracic or neurological symptoms; residual varicose veins; edema; fiber rupture, whether detected during the procedure or afterwards; EHIT; DVT; arteriovenous fistulas; pulmonary embolism; or death.^27^^,^^28^

## DISCUSSION

Development of the ATTA technique was made possible by the conjunction of techniques that are well-established in the literature, emergence, development, and adoption of new technologies, improved understanding of venous system hemodynamics, and the experience acquired during phase four studies.^29^

A review of data in the literature on treatment of varicose tributaries with endolaser identified reports and case series, comparisons with other techniques, and studies on its use for neovascularization of the saphenofemoral junction. However, the steps involved in managing tributaries have not previously been standardized. Development and systemization of the ATTA technique has yielded a proposal for a standardized methodology for treatment of varicose and esthetically unappealing tributary veins in a variety of different areas of the body.^4^

The patient’s personal preferences, advanced age, and physical condition, the surgeon’s experience and the effect and duration of the medications to be used are all factors that must be observed when deciding on whether to treat in an ambulatory or hospital setting.^30^

In this description of a standardized technical proposal, we suggest adjustments to energy levels that are based on clinical practice, with pathophysiological and theoretical foundations, and are supported by recommendations in the literature on venous thermal ablation.

Since this is a pioneering technique, it is essential to conduct prospective, randomized, controlled clinical trials to establish its efficacy and safety in comparison with the established standard treatment.

The protocols presented in this article constitute a suggested technical flow, but do not propose limitations for their use. The final decision falls to the physician responsible for managing each case.

## CONCLUSIONS

The ATTA technique is proposed as a tool for treatment of CVD and esthetically unappealing veins that can be performed in an outpatient setting, suggesting possible extension of the applications for lasers beyond trunk veins to any vein that can be punctured, of any caliber, that are dysfunctional or esthetically unappealing, in a minimally invasive intervention.

Appropriate standardization leading to systematized execution of the technique can shorten the learning curve and enable linear and reproducible results to be achieved.
