# Supplementary material for: Assisted total thermal ablation: presentation of the ATTA technique
Source: J Vasc Bras. 2022 Nov 14;21:e20220048. doi: 10.1590/1677-5449.202200481 (PMC9675071; doi:10.1590/1677-5449.202200481)
Supplement: Translation

# Translation: Ablação térmica total assistida: apresentação da técnica ATTA

**DOI:** 10.1590/1677-5449.202200482

**Authors:** Daniel Amatuzi, Daniel Autran Burlier Drummond, Douglas Poschinger-Figueiredo, Lucas Barbosa-Silva, Julio Cesar Peclat de Oliveira, Marcos Arêas Marques

**Affiliations:** 1 Clínica Vascular ATTA Concept, Maringá, PR, Brasil.; 2 Clínica Daniel Drummond, Rio de Janeiro, RJ, Brasil.; 3 Universidade do Estado do Rio de Janeiro - UERJ, CT Vascular, Rio de Janeiro, RJ, Brasil.; 4 Clínica Patrícia Holderbaum, Canoas, RS, Brasil.; 5 Clínica Peclat, Rio de Janeiro, RJ, Brasil.; 6 Universidade do Estado do Rio de Janeiro - UERJ, Rio de Janeiro, RJ, Brasil.; 7 Universidade Federal do Estado do Rio de Janeiro - UNIRIO, Rio de Janeiro, RJ, Brasil.

**Keywords:** varizes, insuficiência venosa, veia safena, terapia a *laser*, qualidade de vida, estética

## Abstract

O tratamento da doença venosa crônica dos membros inferiores evoluiu de forma exponencial nas últimas décadas. Tais avanços permitiram o desenvolvimento de uma proposta de execução técnica sistematizada para o tratamento de ablação endovenosa com *laser*, a ablação térmica total assistida (ATTA). A técnica propõe um método padronizado de abordagem das veias axiais ou tributárias, varicosas ou inestéticas, de membros inferiores ou outros territórios, em regime ambulatorial ou de hospital-dia. Foram descritos os processos de preparo pré-operatório, marcação detalhada, materiais necessários, acessos venosos, anestesia, cálculo de potência e energia, a técnica de ablação, seguimento e eventos adversos. A ATTA é proposta como uma ferramenta para o tratamento da doença venosa crônica e das veias inestéticas, sugerindo possíveis expansões para as aplicações do *laser*, além dos troncos venosos, para toda veia passível de ser puncionada.

## INTRODUÇÃO

O tratamento da doença venosa crônica (DVC) dos membros inferiores evoluiu de forma exponencial nas últimas décadas, transcendendo das técnicas clássicas para outras menos invasivas. As técnicas endovasculares ablativas apresentam grande destaque, com tendência a desospitalização e maior realização de procedimentos em ambiente exclusivamente ambulatorial. Entre estas técnicas, o *laser* endovenoso (EVLT, do inglês *endovenous laser therapy*) está entre as mais difundidas e versáteis^1^^,^^2^.

Após o primeiro caso de tratamento axial descrito em 1998 por Boné^3^, diversos aprimoramentos e otimizações do método foram realizados; como a evolução das fibras planas para radiais, de menor calibre, o surgimento dos aparelhos geradores de *laser* com melhor perfil na entrega de energia e a posterior expansão de indicações para veias tributárias^4^^,^^5^.

Entre as vantagens das técnicas termoablativas, figuram os menores períodos de convalescência e de necessidade da elastocompressão após o procedimento, resultando em maior comodidade para o paciente. Além disso, há menor lesão aos tecidos perivenosos, menor risco de sangramento e infecção e a possibilidade de realizar o procedimento sem a presença do anestesiologista, com eficácia, segurança e taxa de oclusão equivalentes às de cirurgia convencional^6^^-^9.

Apesar de o EVLT elencar-se como a técnica de escolha para o tratamento das veias axiais do sistema venoso superficial tanto pela Society for Vascular Surgery quanto pela European Society for Vascular Surgery, essas mesmas sociedades recomendam a flebectomia ambulatorial para o tratamento das tributárias varicosas^10^^,^^11^.

A conduta predominante no Brasil é a realização de múltiplas flebectomias em ambiente hospitalar, normalmente sob raquianestesia ou anestesia geral. Como opção de método, tem-se a flebectomia ambulatorial, marcantemente subutilizada, dado que há limitações em casos de doença extensa, frequentemente observados na América Latina^12^.

Descrevemos, neste estudo de inovação, uma proposta de sistematização técnica para a execução do EVLT, a ablação térmica total assistida (ATTA), além dos eventos adversos perioperatórios observados e do perfil de segurança associado à técnica.

## MÉTODOS

A técnica operatória foi descrita de maneira pormenorizada, favorecendo o entendimento dos seus passos e da demanda logística necessária à sua execução, a fim de permitir a sua reprodutibilidade.

A técnica versa sobre o tratamento com *endolaser* das veias tronculares e/ou tributárias, em regime ambulatorial ou de hospital-dia. Para alguns pacientes, existe a necessidade de tratamento complementar durante o seguimento. De forma individualizada, conforme a demanda estética, podem ser utilizados o *laser* transdérmico ou a escleroterapia líquida de forma complementar.

Foram considerados candidatos ao tratamento com a técnica os pacientes portadores de veias insuficientes, dilatadas, tortuosas, varicosas ou inestéticas dos membros inferiores ou de outros territórios, como membros superiores e face. A técnica engloba, além dos eixos safênicos, tributárias que fazem a conexão das veias tronculares com as reticulares e telangiectasias^10^^,^^11^.

Foram consideradas como contraindicações para o tratamento: infecção cutânea ativa na região a ser tratada; risco cirúrgico da American Society of Anesthesiologists (ASA) III ou IV; paciente em uso de anticoagulantes ou estados de hipercoagulabilidade; vigência de ciclo gravídico-puerperal; e histórico de alergia às medicações utilizadas na técnica.

### Protocolo da técnica

Após a obtenção da anamnese e do exame físico, um flebograma (ultrassonografia vascular com Doppler [USD] para o mapeamento venoso detalhado, com registro dos parâmetros de interesse para a realização da técnica ATTA, contendo trajetos, localização e calibres dos vasos que serão abordados, registro de distâncias da parede venosa à pele e da proximidade às estruturas adjacentes) é realizado nos indivíduos candidatos ao tratamento.

1. Marcação detalhada
  - Manutenção da temperatura ambiente em 25 ºC, a fim de evitar a vasoconstrição pelo frio;
  - Registro fotográfico do território a ser tratado em todas as incidências, incluindo imagens com o paciente em ortostase, decúbito ventral e dorsal;
  - Disposição do flebograma previamente realizado;
  - Disposição dos aparelhos de ultrassonografia, fleboscópio e/ou realidade aumentada, objetivando revisar calibres e trajetos de veias tronculares, tributárias e perfurantes; e
  - Marcação das veias de interesse seguida de novo registro fotográfico. O padrão de símbolos sugerido está detalhado na Tabela 1.

**Tabela 1:** Símbolos utilizados na marcação detalhada dos trajetos programados para o tratamento.

**Símbolos**	**Interpretação**
- - - - - - - - - - -	Trajeto das veias safenas insuficientes.
linha tracejada	
. . . . .	Sítios programados para a realização dos botões anestésicos e introdução da agulha de anestesia tumescente ou dos cateteres intravenosos.
ponto	
→ → → → → →	Trajeto programado para progressão dos cateteres, após a punção da veia.
setas direcionais	
+ + +	Sítios programados para a introdução dos cateteres.
cruz

Na primeira etapa, utilizando o flebograma, o paciente é posicionado em decúbito dorsal e ventral e realiza-se a marcação dos trajetos venosos tronculares e/ou tributários a serem abordados, com o auxílio de USD, fleboscópio e/ou aparelho de realidade aumentada.

Nessa etapa, pode-se marcar os possíveis locais de tumescência, com pontos, e de introdução dos cateteres, com pontos e setas (Figura 1).

**Figura 1:** Marcação detalhada da face medial do membro inferior esquerdo. **(A)** Flebograma evidenciando o refluxo ostial das veias safena interna e safena acessória anterior, transmitido às veias arqueadas anterior e posterior; **(B)** Padronização de símbolos sugerida para a realização do procedimento.

A segunda etapa é feita com o paciente em ortostatismo, preferencialmente em plataforma elevada. A marcação é conferida pela ultrassonografia, confirmando os trajetos das veias safenas e os locais para tumescência.

Os pontos de tumescência distam de 5 a 6 cm entre si e acompanham a linha tracejada, longitudinalmente, afastados de 2 a 3 cm dela. Tanto nesses pontos como no sítio proposto para inserção do cateter, o botão anestésico deve ser realizado, conferindo conforto ao paciente. A marcação pode ser realizada em consulta no dia anterior ou logo antes do procedimento.

1. Assepsia, materiais e anestesia

O paciente é posicionado em decúbito dorsal, sendo realizada sedação consciente e analgesia com óxido nitroso. Realiza-se a antissepsia com clorexidina 2% e a colocação dos campos cirúrgicos estéreis após a montagem da mesa com os materiais^13^.

São necessários para a execução da técnica: o gerador de *endolaser* 1.470 nm; fibras radiais 400 μm e/ou 600 μm, sendo a última opcional em caso de calibres maiores da veia safena; conjunto introdutor 6F x 11 cm e/ou cateteres intravenosos 16G ou 14G para punções tanto de tributárias como de safenas; seringas *luer slip* para salinização dos acessos e *luer lock* de 20 mL para solução tumescente; e uma seringa de 10 mL *luer lock* para os botões anestésicos (Figura 2).

**Figura 2:** Exposição dos materiais utilizados para a realização da técnica de ablação térmica total assistida (ATTA).

Confeccionam-se os botões anestésicos, com 0,2 a 0,3 mL de volume por ponto, utilizando solução de 1% a 2% de lidocaína, sem vasoconstritor, observando o tempo de latência de 3 a 5 minutos do anestésico.

1. Acessos

Quando houver indicação de tratamento concomitante das veias axiais e tributárias, um ordenamento lógico é fundamental para a prevenção de vasoconstricção nas veias acessadas. Inicialmente, é garantido o acesso à safena interna, preferencialmente com o introdutor 6F, e, em seguida, são realizados os acessos às tributárias (Figura 3).

**Figura 3:** Membro inferior esquerdo. Observamos a utilização de símbolos padronizados para a técnica. Um conjunto introdutor 6F está inserido na veia safena interna e diversos cateteres/jelcos garantem o acesso às veias tributárias.

São sequencialmente abordadas as faces mediais do pé, da perna e da coxa, seguidas pelas faces laterais do pé, da perna e da coxa. Realiza-se a ablação dessas veias, seguindo a mesma ordem proposta para os acessos e, finalmente, o tratamento da safena magna. De acordo com a indicação do caso, o paciente é posicionado em decúbito ventral e são tratadas as veias safena parva e tributárias posteriores, seguindo o protocolo descrito.

A punção ideal dista 10 mm da veia-alvo e visa tanto a ablação completa do segmento acessado, garantindo espaçamento da pele, quanto a criação de um ponto para ancoragem do cateter, conferindo segurança para as tumescências e as termoablações. Os ângulos entre a agulha e a pele, durante as punções de tributárias, habitualmente serão inferiores a 45º, com progressão paralela à pele.

Utilizando a marcação prévia, sugere-se a realização das punções obtendo as imagens ultrassonográficas em cortes transversais da veia e do cateter intravenoso. A ponta do cateter deve ser mantida no centro da veia, formando o “sinal do alvo”. A progressão do cateter segue a anatomia da veia, navegando sua tortuosidade e extensão desejada para o tratamento. Uma nova punção será necessária quando, devido à angulação, o cateter tocar a parede da veia, interrompendo sua progressão e agregando risco de lesão a ela.

O paciente é posto em posição de Trendelenburg a fim de esvaziar o leito venoso. A salinização do cateter é feita imediatamente após a retirada da agulha, com infusão de 2 a 3 mL de soro fisiológico.

Para o tratamento das tributárias, habitualmente há introdução delicada da fibra radial de 400 μm pelos jelcos até seu ponto máximo de progressão. Mesmo pequenos segmentos de veias devem ser abordados para a obtenção do resultado. Na hipótese do “*heat pipe*”, a transferência direta de calor e o efeito óptico-térmico ocorrem por volta de 6 mm distais à fibra, sendo possível atingir temperaturas de 100 ºC por mais 14 mm, através de bolhas superaquecidas, totalizando uma área de lesão térmica venosa efetiva de até 20 mm além do ponto final de posicionamento do instrumento ablativo^14^.

1. Tumescência

A tumescência tem como objetivos a hidrodissecção, a proteção das estruturas perivenosas, principalmente da pele, e o colabamento da veia a ser tratada, mantendo-a com a menor quantidade possível de sangue na luz, o que reduz as complicações e potencializa os resultados^15^.

A solução anestésica tumescente de lidocaína 0,05%, salina, bicarbonato de sódio e adrenalina é utilizada em todos os casos, ainda que o paciente tenha sido submetido ao bloqueio espinhal ou à anestesia geral, proporcionando conforto no pós-tratamento^16^^,^^17^.

O volume e a concentração de lidocaína podem ser ajustados de acordo com o peso do paciente e com a extensão da área a ser tratada, respeitando os limites da dose tóxica da substância. Extremidades corporais exibem maior sensibilidade à dor, bem como áreas adjacentes às veias perfurantes, devido à proximidade aos feixes vasculonervosos.

Um dos objetivos da tumescência é obter um halo de 10 mm de raio circunjacente à veia-alvo, utilizando como referência a distância da pele à parede anterior da veia, o que nem sempre é possível. Caso o halo tumescente seja inferior a 8 mm, o cálculo da potência a ser usada é afetado (Figura 4)^15^^,^^16^.

**Figura 4:** Aquisição ultrassonográfica durante a anestesia tumescente. Observamos na imagem: **(A)** a fibra de *endolaser*; **(B)** a agulha de anestesia tumescente; e **(C)** o halo de tumescência, conferindo o distanciamento da parede anterior da veia até a pele.

Na abordagem de regiões com diversas punções próximas, a fim de reduzir o risco de complicações, recomenda-se a utilização de solução tumescente em baixa temperatura, aproximadamente 4 °C, e maior intervalo temporal entre ablações sucessivas, evitando a criação de um gradiente crescente de temperatura.

1. Cálculo da potência e energia ablativa

Esta é uma etapa primordial do procedimento. A termoablação em si gera receios quanto à energia a ser utilizada devido ao risco de complicações, como lesões térmicas da pele e outras estruturas perivenosas. São variáveis relevantes o comprimento de onda do *laser*; o modo de entrega da energia; a velocidade de tração e tipo de fibra; a densidade de energia endovenosa linear (LEED, do inglês *linear endovenous energy density*); e a potência nominal^6^^,^^18^.

O *laser* diodo de 1.470 nm possui maiores vantagens na termoablação endovenosa, com trabalhos demonstrando melhor perfil de entrega de energia em relação a outros comprimentos de onda e superioridade nos resultados do tratamento^4^^,^^19^.

Os geradores de *laser* podem entregar a energia luminosa em modo pulsado ou contínuo. O último confere maior facilidade na quantificação da energia total por segmento de veia tratada, ou de superfície, sendo o método de eleição para a ATTA. Quanto à entrega de potência, há variação de 1 a 40 watts (W), conforme o fabricante da unidade^20^.

Para o tratamento dos troncos venosos principais, é ampla a discussão sobre o melhor modo de calcularmos a energia ideal para a efetiva termoablação. Contudo, a maneira mais difundida em nosso meio, proposta desde 2005, é o cálculo energético por LEED, tendo como base a fórmula simplificada LEED = P x T / centímetro linear de veia, em que P é a potência e T, o tempo^21^.

Temos, portanto, duas variáveis a manejar no cálculo da LEED: a potência nominal, em W, e a velocidade de tração, em milímetro por segundo. Visando padronizar a técnica, fixamos a velocidade de tração em 1 mm/s^21^.

Na técnica ATTA, utilizamos um protocolo detalhado para o ajuste da potência. Como a velocidade de tração é fixa em 1 mm/s, o LEED final, em J/cm, será o valor nominal da potência multiplicado por dez.

O cálculo de potência deve ser obtido no momento da programação do procedimento, normalmente após se realizar o flebograma, o que facilita sua execução e dá maior agilidade à intervenção (Tabela 2). Aplicativos para dispositivos móveis, como o *ATTA* *Score*, podem simplificar a realização desse cálculo (Figura 5). A medição do raio do halo tumescente é a variável, com impacto no cálculo, e deve ser definida durante o procedimento.

**Tabela 2:** Variáveis implicadas para o estabelecimento do cálculo de potência.

**Variáveis**	Energia adicionada*
Energia inicial	2,5
Índice de massa corpórea	
• > 30 kg/m^2^	0,5
• < 30 kg/m^2^	0
Classificação CEAP†	
• 1-3	0
• 4-6	1,0
Topografia das varizes	
• Tributárias acima do joelho	0,5
• Tributárias na perna	0
• Tributárias no tornozelo e/ou abaixo (exceção)	0
Diâmetro da veia-alvo	
• Até 3 mm	0,5
• 3,1-5 mm	1,0
• 5,1-10 mm	1,5
• > 10 mm (exceção)	0
Halo de tumescência	
• < 8 mm	-1,0
• > 8 mm	0

* Energia em watts (W);

† Estadiamento clínico (C) da classificação clínica, etiológica, anatômica e patológica (CEAP).

**Figura 5:** Interface do aplicativo auxiliar, *ATTA Score*, de cálculo da potência a ser utilizada na intervenção. IMC = índice de massa corpórea; CEAP = classificação clínica, etiológica, anatômica e patológica; alt = alterações.

Iniciamos com a potência de 2,5 W, seguida por acréscimos ou subtrações, de acordo com as variáveis a seguir.

- Índice de massa corpórea (IMC). Quando igual ou maior do que 30 kg/m^2^, adicionamos 0,5 W de potência, dada a maior chance de recanalização após EVLT^22^;
- Classificação clínica, etiológica, anatômica e patológica (CEAP). Incrementamos em 1,0 W a potência dispensada a pacientes C4, C5 ou C6 - situações em que foi demonstrada maior necessidade de reintervenções^23^;
- Topografia do vaso-alvo. Varicosidades em região de coxa e joelho são tratadas com acréscimo de 0,5 W;
- Diâmetro da veia. É uma variável de grande relevância para a execução da técnica. Em veias com calibre menor do que 3 mm, soma-se 0,5 W. Entre 3 e 5 mm de diâmetro, acrescenta-se 1,0 W e, acima de 5 mm, adiciona-se 1,5 W na potência^24^; e
- Anestesia tumescente. Apresenta papel importante na prevenção de complicações, pela obtenção do halo tumescente.

Nas tributárias em região infragenicular, não há incremento ou subtração no cálculo da potência. Varizes em tornozelos e pés configuram uma exceção ao cálculo, pois exigem diferentes potências e velocidades de tração. É recomendada a abordagem com maior experiência técnica, dado que territórios mais distais carregam maior risco de complicações.

As veias perfurantes necessitam maior quantidade de energia para a adequada termoablação. Habitualmente, somamos 1,0 W na potência a ser utilizada após aplicarmos a tabela de cálculo^25^.

1. Técnica de ablação

Após calculada a potência e introduzida no gerador de *laser*, prosseguimos com a retração parcial da bainha do cateter/jelco e com o disparo sequencial do *laser*. A tração deve manter constância de 1 mm/s, sincronizando o movimento de recuo da fibra com a emissão sonora de um bipe por segundo, função disponibilizada por alguns aparelhos geradores. Durante a termoablação, uma das mãos realiza a tração da fibra, enquanto a outra maneja o transdutor, observando os sinais ultrassonográficos de resposta tecidual, bem como a possível necessidade de complementação da tumescência. Nos últimos dois centímetros, torna-se mais prático utilizar a compressão manual, mantendo o colabamento da veia e controlando a temperatura local. Os últimos passos consistem na retração do conjunto de fibra e bainha do cateter e na compressão digital por no mínimo 30 segundos, ou até se obter a hemostasia^14^.

No controle ecográfico imediato, nota-se oclusão da veia tratada e ausência de fluxo a USD. De forma análoga ao tratamento dos troncos safênicos, eventualmente conseguimos perceber o “sinal da pérola” na imagem transversa, a linha branca no plano longitudinal, bem como uma pequena sombra acústica quando há formação de bolhas^16^.

Realiza-se curativo com compressão excêntrica nos sítios das punções, seguido de elastocompressão, habitualmente feita com meias de 35 mmHg por 24 h, com o intuito de diminuir edema e hematomas. O paciente é orientado a utilizar a própria meia elástica de 35 mmHg ou de menor compressão, de 20 a 30 mmHg, por 1 a 3 semanas no período diurno. O registro fotográfico pós-operatório permite o estabelecimento de uma referência para o seguimento (Figura 6)^26^.

**Figura 6:** Registros fotográficos pré e pós-operatórios de pacientes submetidos à técnica de ablação térmica total assistida (ATTA).

Com a metodologia descrita, a técnica ATTA pode ser aplicada em outras áreas do corpo, como em veias inestéticas de mão e da fronte. Tais zonas demandam maior precisão na execução, cuidados individualizados e observância à anatomia durante a tumescência e termoablação.

1. Seguimento ultrassonográfico

É recomendada a realização de uma USD de controle entre o 3º e o 7º dia da realização do procedimento, excluindo a possível ocorrência de trombose venosa profunda (TVP) ou trombose induzida pelo calor (EHIT, do inglês *endothermal heat induced thrombosis*), principalmente nos pacientes tratados nos eixos tronculares safênicos ou que apresentem comunicações com veias perfurantes^27^.

### Perfil de segurança e eventos adversos

Os eventos adversos observados incluíram dor, tromboflebite, equimose, hipercromia, cordão endurado, lesão neurológica periférica, com disestesia transitória, e queimadura de primeiro grau da pele. Tais eventos são observados em outras técnicas termoablativas, e casos sintomáticos formam manejados com anti-inflamatório não esteroide por 5 dias. À exceção da hipercromia e do cordão endurado residual, os sintomas correspondentes aos demais eventos adversos estavam ausentes após 30 dias^7^^,^^16^.

Ainda que tenham sido descritas na literatura, não foram observadas em nossa experiência ocorrências de mal-estar; *rash*; alergia; constrição cervical; tosse; sintomas torácicos ou neurológicos; varizes residuais; edema; ruptura da fibra, flagrada no momento do procedimento ou posteriormente; EHIT; TVP; fístulas arteriovenosas; embolia pulmonar; ou óbito^27^^,^^28^.

## DISCUSSÃO

A conjunção das técnicas bem estabelecidas na literatura, o surgimento, a evolução e a popularização das novas tecnologias, a melhor compreensão da hemodinâmica do sistema venoso e a experiência adquirida com os estudos de fase quatro permitiram o desenvolvimento da técnica ATTA^29^.

A revisão na literatura de dados sobre o tratamento de tributárias varicosas com *endolaser* contém relatos e séries de casos, comparações com outras técnicas e seu uso para neovascularização de crossa. Entretanto, não há padronização das etapas envolvidas na abordagem das tributárias. A elaboração e sistematização da técnica ATTA propõe uma metodologia padronizada para o tratamento de veias tributárias varicosas e inestéticas em diversas topografias^4^.

As preferências pessoais do paciente, a idade avançada, a condição física, a experiência do cirurgião e o efeito e a duração das medicações a serem utilizadas são fatores que devem ser observados na opção pelo regime ambulatorial ou hospitalar^30^.

Nessa descrição de proposta técnica padrão, sugerimos ajustes energéticos baseados na prática clínica, com embasamento fisiopatológico e teórico, pautados por recomendações da literatura sobre termoablação venosa.

Por se tratar de técnica vanguardista, é imperioso que sejam realizados estudos clínicos randomizados, prospectivos e controlados para o estabelecimento da sua eficácia e segurança frente ao padrão estabelecido de tratamento.

Os protocolos contidos neste estudo figuram uma sugestão de fluxo técnico, não propondo uma limitação para sua execução. A decisão final cabe ao médico responsável pela condução dos casos.

## CONCLUSÃO

A técnica ATTA é proposta como uma ferramenta para o tratamento da DVC e das veias inestéticas, factível em ambiente ambulatorial, sugerindo possíveis expansões para as aplicações do *laser,* além dos troncos venosos, para toda veia passível de ser puncionada, de qualquer calibre, insuficientes ou inestéticas, sob intervenção minimamente invasiva.

A padronização apropriada, levando a execução sistematizada da técnica, pode abreviar a curva de aprendizagem, favorecendo a obtenção de resultados lineares e reprodutíveis.
